# Gene regulatory and gene editing tools and their applications for retinal diseases and neuroprotection: From proof-of-concept to clinical trial

**DOI:** 10.3389/fnins.2022.924917

**Published:** 2022-10-20

**Authors:** Halit Yusuf Altay, Fatma Ozdemir, Ferdows Afghah, Zeynep Kilinc, Mehri Ahmadian, Markus Tschopp, Cavit Agca

**Affiliations:** ^1^Molecular Biology, Genetics and Bioengineering Program, Sabanci University, Istanbul, Turkey; ^2^Department of Ophthalmology, Cantonal Hospital Aarau, Aarau, Switzerland; ^3^Department of Ophthalmology, Inselspital, Bern University Hospital, University of Bern, Bern, Switzerland; ^4^Nanotechnology Research and Application Center (SUNUM), Sabanci University, Istanbul, Turkey

**Keywords:** CRISPR-Cas, rare diseases, retina, gene therapy, TALE, zinc finger, antisense oligonucleotides

## Abstract

Gene editing and gene regulatory fields are continuously developing new and safer tools that move beyond the initial CRISPR/Cas9 technology. As more advanced applications are emerging, it becomes crucial to understand and establish more complex gene regulatory and editing tools for efficient gene therapy applications. Ophthalmology is one of the leading fields in gene therapy applications with more than 90 clinical trials and numerous proof-of-concept studies. The majority of clinical trials are gene replacement therapies that are ideal for monogenic diseases. Despite Luxturna’s clinical success, there are still several limitations to gene replacement therapies including the size of the target gene, the choice of the promoter as well as the pathogenic alleles. Therefore, further attempts to employ novel gene regulatory and gene editing applications are crucial to targeting retinal diseases that have not been possible with the existing approaches. CRISPR-Cas9 technology opened up the door for corrective gene therapies with its gene editing properties. Advancements in CRISPR-Cas9-associated tools including base modifiers and prime editing already improved the efficiency and safety profile of base editing approaches. While base editing is a highly promising effort, gene regulatory approaches that do not interfere with genomic changes are also becoming available as safer alternatives. Antisense oligonucleotides are one of the most commonly used approaches for correcting splicing defects or eliminating mutant mRNA. More complex gene regulatory methodologies like artificial transcription factors are also another developing field that allows targeting haploinsufficiency conditions, functionally equivalent genes, and multiplex gene regulation. In this review, we summarized the novel gene editing and gene regulatory technologies and highlighted recent translational progress, potential applications, and limitations with a focus on retinal diseases.

## Introduction

**FIGURE 1:**
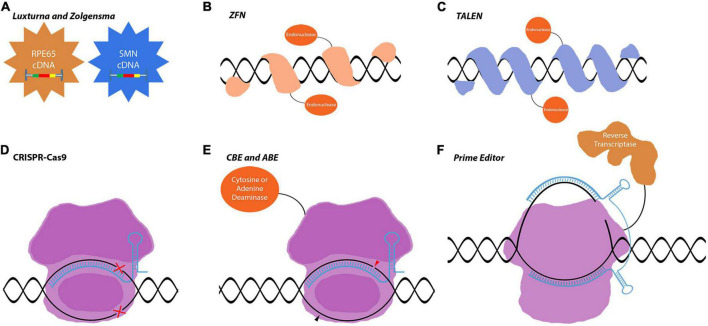
Gene editing tools used in gene replacement and gene correction techniques. **(A)** Luxturna and Zolgensma are AAV-based drugs that replace the mutant gene-of-interest with the wild-type copy. **(B–F)** Gene correction relies on more intricate tools to edit the genome and precisely correct mutations in the target gene. This enables the correction of mutations in large genes that do not fit into viral vectors. **(B–D)** These first-generation gene correction tools rely on DNA-repair mechanisms to correct mutations. **(E,F)** CBEs, ABEs, and prime editors are very precise gene editing tools that can correct point mutations. Images are modified from crystal structures of proteins (Protein Data Bank: https://www.rcsb.org/search/advanced/sequence).

Nowadays, medicine is in a state of transformation where novel approaches, including gene therapies, for untreated diseases are becoming available. Gene therapies that are tailored for specific diseases are growing in number in the last decade. One of the research areas that gained considerable attention in gene therapy is retinal degenerative diseases. The first successful gene therapy approach for an inherited retinal disorder is voretigene neparvovec (Luxturna). Voretigene neparvovec (VN) is an AAV2-mediated gene replacement therapy for the treatment of Leber congenital amaurosis (LCA) associated with biallelic mutations in *RPE65*. It delivers wild-type cDNA of the *RPE65* gene, which is critical for the visual cycle and is shown to improve the visual function of patients (Maguire et al., 2008). Onasemnogene abeparvovec (Zolgensma) is another gene replacement therapy that delivers the functional copy of survival of motor neuron 1 (SMN1) gene using AAV9 for the treatment of spinal muscular atrophy (SMA), a devastating neuromuscular disease (Hoy, 2019; Day et al., 2021; Figure 1A). These two pioneering therapies are mainly gene replacement therapies where a mutated gene is compensated by the delivery of wild-type cDNA, referred to as first-generation gene therapies. While the therapeutic logic behind these therapies is quite straightforward, several efficiency steps had to be overcome to be approved by governmental agencies.

Gene replacement, gene editing, and other gene regulatory treatments for ophthalmology are advancing in terms of numbers, complexity, and approval of therapies. It is because the eye is better suited for translational research on gene therapy due to its remarkable properties compared to other organs. It is small and easily reachable to perform non-invasive or microsurgery techniques with ease of application. For instance, the small size of the eye is beneficial to reduce the effective therapeutic dosage. Moreover, the eye is a rather isolated organ, which minimizes the systemic effects of treatment. Finally, due to the accessibility of the eye, the administering of therapeutics and assessing their effects through imaging can be accomplished quite readily (Ong et al., 2019; Ziccardi et al., 2019).

**FIGURE 2:**
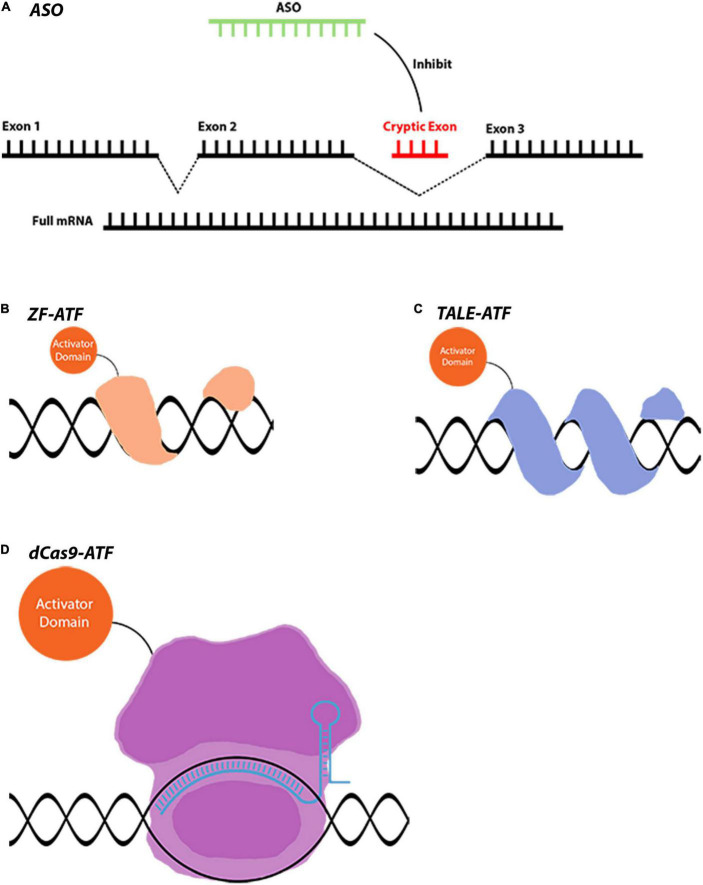
Gene regulation tools that are used for indirect gene therapies. **(A)** AON-mediated splicing correction is used in LCA and many other diseases. AONs can act in different ways such as gene silencing and miRNA modulation. **(B–D)** ATFs are novel tools that can downregulate or upregulate a gene to achieve a therapeutic effect. Conjugation of different domains to achieve varying levels of gene regulation makes these tools extremely versatile. Images are modified from crystal structures of proteins (Protein Data Bank: https://www.rcsb.org/3d-sequence).

AAVs are the main delivery vehicles for ophthalmological gene therapies. They are comparably safer than lentiviruses or adenoviruses as they stay mostly episomal (Penaud-Budloo et al., 2008; Schön et al., 2015). Targeting different cell populations is achievable by using different AAV serotypes or through the directed evolution of new capsid proteins. AAV serotypes have been studied in detail with confirmed safety profiles in retinal applications (Schön et al., 2015). While AAVs have such advantageous properties, they are inadequate in certain aspects that limit ocular gene therapy applications. Their capacity is only 4.8 kb which limits the cDNA delivery approach to small genes. Mutations in large inherited retinal dystrophy (IRD) causing genes, such as *ABCA4* and *MYOC7A*, require complex tools and therapeutic strategies to be corrected *in vivo*. With the increased approval of first-generation therapies, more complex therapies will be developed exploiting gene regulation and gene editing tools for the treatment of IRDs. These second-generation/more advanced therapies will be using novel tools, which are already at the translational level and the subject of proof-of-concept studies or early clinical trials (Figures 1, 2). The most well-known of these tools is CRISPR-Cas9 which is currently a fundamental tool for both basic and translational research (Figure 1D). CRISPR-Cas9 is an antiviral defense mechanism found in certain bacteria and archaea. This system engraves parts of invader DNA into the host’s genome to detect and destroy any further invasions (Makarova and Koonin, 2015).

Usage of CRISPR-Cas9 in IRDs allows for correcting the existing mutations and protecting the retinal neurons. Recently, several CRISPR-Cas9-associated gene editing tools have been developed such as dCas9-nickases, base editors, and prime editors (Komor et al., 2016; Satomura et al., 2017; Anzalone et al., 2019; Figures 1D–F). Novel CRISPR-Cas proteins were identified with diverse functions as well. With the discovery of CRISPR-CasRx, it is also now possible to do gene editing at the mRNA level further increasing the therapeutic possibilities (Konermann et al., 2018).

Gene regulatory approaches, in which the genome remains unedited and only gene expression is modified, are also critical to protect retinal neurons (Figure 2). As in the case of antisense oligonucleotides, eliminating pathogenic mRNA or correcting the splicing defects can rescue the detrimental effects of mutant proteins on retinal neurons. Moreover, activating survival pathways or functionally equivalent genes using artificial transcription factors are additional neuroprotective approaches for the survival of retinal neurons. Considering this, neuroprotection might be necessary together with gene correction therapies for the survival of the remaining cells in neurodegenerative diseases (Pardue and Allen, 2018).

Due to the increasing number of gene editing and gene regulatory tools, it may be difficult to keep track of advancements and determine the most effective approach for each tool, as they all have different advantages and disadvantages. These advancements regarding the novel approaches already accelerated the development of further gene therapies. Moreover, several new treatments are expected to be approved in the upcoming years. As of 2025, 10–20 gene therapies are expected to be approved each year. This will be a game-changer and will restructure medicine in various aspects.

This review provides an in-depth insight into gene therapy and neuroprotection in the retina for researchers and clinicians. The necessary techniques and tools for novel gene therapies are thoroughly discussed with the focus on gene regulatory and gene editing tools such as CRISPR-Cas9, catalytically inactive/dead cas9 (dCas9), Transcription activator-like effectors (TALE), zinc finger (ZF), CasRx, Transcription activator-like effector nucleases (TALEN), Zinc-finger nuclease (ZFN), base editors, prime editors, and antisense oligonucleotides (AON) (Figures 1, 2). Ultimately, this article should provide novel knowledge on gene delivery tools and to some extent animal models that are required for future proof-of-concept studies.

## Ongoing clinical trials for retinal gene therapies

**FIGURE 3:**
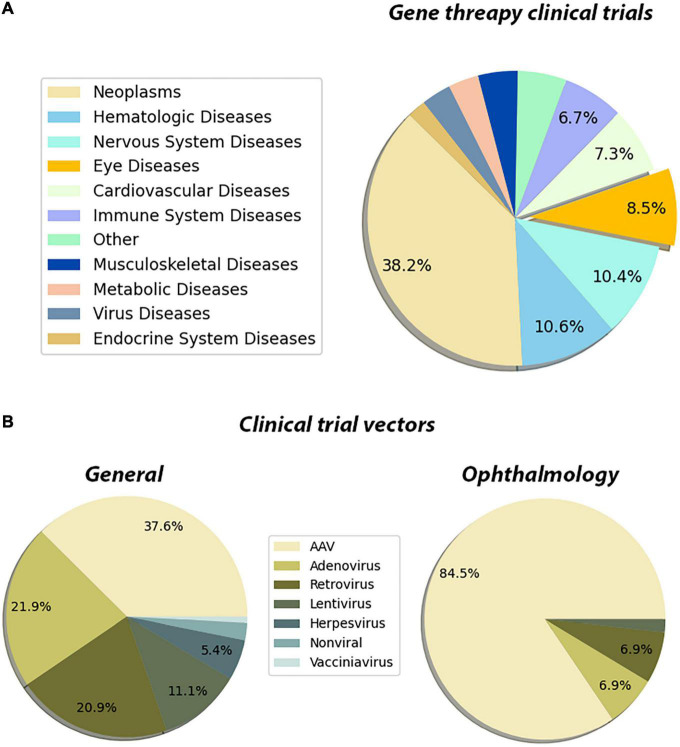
Pie chart distribution of disease categories and vectors used in gene therapy clinical trials. **(A)** The distribution of the disease categories targeted by the clinical trials that are using gene therapy methods. The metadata of the gene therapy trials has been downloaded from the ClinicalTrials database. The Medical Subject Headings (MeSH) thesaurus has been produced by the National Library of Medicine and serves as a hierarchically organized vocabulary for indexing, cataloging, and searching for biomedical and health-related information (Dhammi and Kumar, 2014). In the tree structure of the MeSH thesaurus, each term has a code indicating the parent groups of the term. By using the “Diseases” categories in the MeSH tree, the clinical trials have been assigned to one of the categories. The assignments have been made by matching the MeSH terms registered in the clinical trial’s entry, with the disease category in the MeSH tree. For entries that have been registered to multiple disease categories, a priority list has been created manually. These clinical trials have been assigned to the disease type which has a higher priority in the created list. The subcategories of the eye diseases have been retrieved from the MeSH terms section of the trial’s page and the bar graph showing the distribution of such subcategories has been plotted. The entries which are associated with multiple subcategories have been assigned to the term which has a higher priority in the created list. **(B)** The distribution of the vectors used in the gene therapy clinical trial. A list of keywords for the vectors that have been used in the gene therapy methods has been created. By using the API (Application Programming Interface) of ClinicalTrials.gov, each keyword has been searched along with the “gene therapy” keyword. In these search operations, the database searches the given terms with their synonymous representations. Through the search operations, the distribution of vectors that have been used in clinical trials has been retrieved. Then the same search has been repeated by adding the keyword “eye diseases.” Through this search operation, the distribution of the used vectors in the gene therapy clinical trials targeting eye diseases has been retrieved. The whole data processing and plotting procedure have been automatized by a custom Python script. Data is registered on ClinicalTrials.gov as of 26/02/2022.

The innate properties of the eye make it an ideal target for gene therapies. This is also reflected in the FDA clinical trials database^1^ as well. Our meta-analysis showed that the neoplasm, hematologic, and the diseases of the nervous system lead the gene therapy field which is followed by eye disease applications (Figure 3A). Out of all gene therapy clinical trials, eye diseases occupy approximately 8%, and the eye is the sole organ to have such a large portion of clinical trials using gene therapy applications (Figure 3A). Another more prominent fact for retinal gene therapy clinical trials is the domination of gene replacement therapies for retinal diseases. More than 60 clinical trials for eye disease are gene replacement therapies. This is most likely due to the monogenic nature of the majority of inherited retinal diseases. There are several *in vivo* studies using novel gene regulatory and gene editing tools for retinal diseases (Table 1). Despite having growing numbers of proof-of-concept studies (Table 1), CRISPR-Cas9 and associated tools have only been used in very few clinical therapies. This already suggests that we are still in the early phases of the gene therapy era and these novel therapies will be in clinical trials in the upcoming years.

**TABLE 1:** Preclinical and clinical studies using gene correction and gene regulatory tools.

	Available gene therapy approaches	Disease	Applications (drugs, clinical trials, proof-of-concept)	Mutated genes
Gene Correction Therapy	CRISPR-Cas9	LCA10	Gene correction (Ruan et al., 2017; Maeder et al., 2019)	*CEP290*
		Glaucoma	Gene correction (Jain et al., 2017; Wu et al., 2020)	*MYOC*
				*AQP1*
		AMD	Knockdown (Ling et al., 2021)	*VEGFA*
	ZFN	RP	Knockout (Greenwald et al., 2010; Overlack et al., 2012)	*Rho*
		Usher syndrome		*USH1C*
	CasRX	AMD	Knockdown (Zhou et al., 2020a,b)	*VEGFA*
		Induced retinal degeneration		*Ptbp1*
	Base Editing	LCA2	Gene correction (Suh et al., 2021)	*RPE65*
	Prime Editing	LCA2	Gene correction (Jang et al., 2021)	*RPE65*
Gene Regulation Therapy	Antisense Oligonucleotides (AONs)	AMD	Knockdown (NCT03815825, NCT04014335)	Complement factor B
		Neovascular glaucoma	Knockdown (NCT02947867)	*VEGFA*
		LCA10	Splicing correction (NCT04855045, NCT03913143)	*CEP290*
		RP	Knockdown (NCT04123626)	*Rho*
		Usher syndrome	Exon skipping (NCT05158296, NCT03780257, NCT05085964, NCT05176717)	*USH2A*
	CRISPR-dCas9	RP	Gene transactivation (Böhm et al., 2020)	*M-opsin & S-Opsin*
	ZFP	RP	Knockdown (Marrocco et al., 2021)	*Rho*

A detailed summary of current preclinical and clinical trials using gene regulatory and gene correction tools are listed based on literature research using clinicaltrials.gov and pubmed.gov as of 20/04/2022.

Another important feature of the existing clinical trials is the choice of the delivery vector. There are now several available viral and non-viral-based vectors for gene therapy applications. With the improvement of the safety profile of AAVs and their widespread usage in clinical trials, AAVs become one of the major vectors for gene therapy applications. While AAVs are the go-to delivery methods for all gene therapy applications (37%) (Figure 3B), they are especially dominating in the eye diseases category (84%) (Figure 3B). This is due to reduced immunogenicity, minimal genome integration and a wide array of capsids of AAVs. Since retinal neurons are non-dividing cells, AAV-based treatments are long-lasting due to episomal maintenance of AAV genomes in the nucleus (Penaud-Budloo et al., 2008; Schön et al., 2015). Therefore, the benefits of AAV-based gene therapy for retinal diseases are maintained for at least several years. Further advancements in non-viral delivery tools nanoparticles and exosomes are also becoming more and more available and provide much safer alternatives (Gee et al., 2020; Wei et al., 2020).

## CRISPR-Cas systems in retinal gene therapy

The discovery of CRISPR-Cas proteins marks a new era in science. The practicality of this system, combined with its adaptability to numerous situations and reduced cost, has made it a great choice in fields ranging from genetics to bioimaging. In nature, CRISPR-Cas systems are elements of bacterial and archaeal adaptive immunity against invasive mobile genetic elements (MGEs) (Makarova and Koonin, 2015). CRISPR-Cas systems rely on the ability of CRISPR-associated endonuclease (Cas) to cleave invading MGEs. Native forms of CRISPR-Cas systems require CRISPR RNA (crRNA) to recognize the target sequence from invader MGEs. crRNA recognizes a target sequence by identifying nearby proto-spacer adjacent motifs (PAMs) conserved in MGEs. In the case of a recognition event, the invader genetic elements are silenced through cleavage. Integration of invader DNA sequences into the CRISPR loci enables immune memory of past infections (Kim et al., 2015). The diversity of these prokaryotic immune system components makes them suitable for diverse applications such as gene editing and molecular diagnostics. The versatility and cost-effectiveness of this system made it favorable for gene editing for inherited retinal diseases as well. From gene knockout and RNA-editing to artificial transcriptional networks, CRISPR-Cas systems offer countless possibilities for future therapeutics for IRDs.

### Applications of CRISPR-Cas9 system in retinal diseases

Cas9 protein belongs to the class 2 type II CRISPR family, and it is the most well-characterized Cas protein. Native Cas9 systems require *trans-*activating CRISPR RNA (tracrRNA) to mediate the complex formation between CRISPR RNA (crRNA) and Cas9 protein, while tracrRNA and crRNA functions are merged to form a single guide RNA (sgRNA) in recombinant CRISPR-Cas9 systems (Pickar-Oliver and Gersbach, 2019). Cas9 has been adapted to many translational studies, and Streptococcus pyogenes Cas9 (SpCas9) is the most extensively studied variant (Figure 1D). CRISPR-Cas9 is a strong tool for the development of therapies against IRDs that have hundreds of candidate genes, with several different mutations on some genes such as the rhodopsin gene (Daiger, 2022). As the majority of IRDs are monogenic syndromes, they are ideal targets for gene correction when the delivery of a complete cDNA is not applicable or desired. Several proof-of-concept studies are using CRISPR-Cas9 mediated gene correction for the treatment of IRDs (Table 1).

Recently, the CRISPR-Cas9 system has been used by Ruan et al. in Leber congenital amaurosis type 10 (LCA10), a subtype of one of the most severe groups of inherited retinal diseases called LCA (Ruan et al., 2017). Mutations in *CEP290* cause LCA10, the most common subtype of LCA (den Hollander et al., 2006). CEP290 protein functions in the transition zone of the rod photoreceptors and mutations in this gene result in ciliopathy with a severe retinal degenerative phenotype (Cideciyan and Jacobson, 2019). IVS26 (c.2991+1655A > G mutation in intron 26) is the most frequent mutation in the *CEP290* gene, causing LCA10. It produces a splice-donor site, a cryptic exon of 128-bp, competing with the normal splice-donor site that results in a premature stop codon in over half of the transcripts (den Hollander et al., 2006). For the correction of mutations in the *CEP290* gene, some obstacles are difficult to overcome with gene replacement. The CEP290 coding sequence is over 7 kb; thus, AAV delivery of the gene replacement therapy is inefficient due to the 4.8 kb size limitation of AAVs. Hence, correction of the aberrant splicing is a more desirable therapeutic strategy in LCA10. In this study, AAV5 was chosen as it showed a specific tropism toward photoreceptor cells, enabling more efficient delivery into photoreceptor cells compared to AAV2 (Lotery et al., 2003). Moreover, including the human rhodopsin kinase 1 (hGRK1) promoter in the vector enhances expression in photoreceptor cells (Khani et al., 2007). Dual AAV5 vectors, one vector with SpCas9 and another with gRNAs, were delivered *via* subretinal injection. Afterward, next-generation sequencing (NGS) was performed on four treated retinas. The dual AAV5 CRISPR-SpCas9 system achieved 20% deletion of IVS26 mutation on average (Ruan et al., 2017). Following these findings, a PAM recognition site and sgRNA sequences were incorporated into the SpCas9 plasmid to attain self-inactivation. This ensured that the CRISPR-SpCas9 system would self-deactivate after the mutation was corrected. The self-limiting SpCas9 system significantly reduced mutant *Cep290* levels while increasing wild-type *CEP290* expression in mutant HEK293T cells (Ruan et al., 2017), suggesting that the dual vector system has higher therapeutic potential.

In a similar study targeting *Cep290*, Maeder and coworkers used a single AAV5 vector to deliver a CRISPR-SaCas9 system for the treatment of LCA10 disorder as a proof-of-concept (Maeder et al., 2019). They used a dual gRNA-directed CRISPR-SaCas9 system in which AAV5 genome integration provided insertions and at the same time, the SaCas9 double-stranded break activity resulted in indel, deletion, and inversion events. The short size of the SaCas9 enabled the delivery of the whole sequence with a single AAV5 vector. The CRISPR-Cas9 construct reduced the mutant *Cep290* expression in patient-derived fibroblasts. In addition, the outer nuclear layer-specific expression of this construct was shown in retinal explants due to the presence of GRK1 promoter and AAV5 tropism. Maeder et al. performed NGS on *in silico* predicted off-target sites and they found no off-target activity in the predicted sites. They started their proof-of-concept studies using the humanized LCA10 mouse model, the HuCEP290 knock-in mouse. They tested two different doses (10^12^ vg^–1^ and 10^13^ vg^–1^) and performed a 9-month follow-up. The two dose groups of 10^12^ vg^–1^ and 10^13^ vg^–1^ reached the same editing levels (∼21%) at week 6 and week 2, respectively. The editing rates stayed the same afterward. They estimated that rescuing 10% of the foveal cones should provide a visual improvement to subjects and they showed that a dose range between 3 × 10^11^ vg^–1^ and 3 × 10^12^ vg^–1^ is ideal for providing a visual improvement. Furthermore, the dosage of AAV was determined in a humanized IVS26 mice model (HuCEP290) and cynomolgus monkeys. Cynomolgus monkeys were used for the final dose assessment before human clinical trials due to the presence of macula, and a rod-to-cone ratio closer to humans in contrast to mice. They reached the required 10% efficiency of foveal cone rescue required for the improvement of vision in their preclinical studies. Following their successful findings, they became the first group to perform a human clinical trial phase I/II utilizing CRISPR-Cas9 in ophthalmology (NCT03872479).

Developing a treatment for monogenic disorders can enable researchers to focus on a handful of targets while multifactorial disorders such as Alzheimer’s and Parkinson’s disease can be quite difficult to develop a treatment for. Glaucoma is a multifactorial disease that is the second leading cause of blindness worldwide. CRISPR-mediated gene correction for primary open-angle glaucoma (POAG), the most common type of glaucoma, was demonstrated in a proof-of-concept study (Jain et al., 2017). Glaucoma is caused by increased intraocular pressure (IOP) that results in retinal ganglion cell (RGC) degeneration. The resistance against aqueous humor outflow by trabecular meshwork (TM), where aqueous humor is drained, causes an increase in IOP. Previously, an animal model for POAG was generated by introducing the human *MYOC* gene containing the Y437H mutation into mice (Zode et al., 2011). Mutant myocilin aggregates in the TM, causing endoplasmic reticulum stress and increased expression of unfolded protein response markers. This affects the normal function of TM cells and has phenotypic outcomes like increased IOP pressure, retinal ganglion cell, and optic nerve degeneration. To eliminate the negative effects caused by the mutant transgene in *Tg-MYOC*^Y437H^** mouse, *MYOC^Y437H^* was targeted with a CRISPR-SpCas9/gRNA complex *via* Adenovirus type-5 (Ad5-crMYOC). Interestingly, a decrease in *MYOC^Y437H^* reduced both IOP levels and the unfolded protein response marker expression. Similarly, increased secretion of wild-type mouse MYOC was also shown in aqueous humor samples. Furthermore, Ad5-crMYOC was tested on human anterior segment cultures, and a reduction of *MYOC^Y437H^* mRNA and increased secretion of wild-type MYOC were reported. These results indicate that targeted inhibition of the mutant *MYOC* gene can indirectly increase the secretion of wild-type MYOC while rescuing the POAG phenotype.

Glaucoma as a multifactorial disease can be caused by many different genes. Some of them cause an abnormal increase in IOP levels and an increased IOP level is considered the biggest risk factor to develop glaucoma (Ocular Hypertension Treatment Study Group et al., 2007). Thus, it can be a better strategy to target the IOP instead of correcting several mutations in different genes. *Aquaporin 1* (*Aqp1*) is a water channel protein that is involved in the ciliary epithelium and TM network to regulate the aqueous humor secretion. Inhibition of *Aqp1* expression was shown to decrease IOP levels significantly *in vivo* (Schey et al., 2014). This property was used in a recent study by targeting *Aqp1* in ciliary epithelium with CRISPR-SaCas9. It was shown that unilateral intravitreal delivery of SaCas9-*Aqp1* (2 × 10^10^ vg) into C57BL/6J mice reduced the IOP by 22% compared to contralateral eyes. The potential therapeutic usage of this system was further evaluated in experimental mouse models of glaucoma. A significant decrease in IOP levels was reported in both the corticosteroid-induced ocular hypertension and the more severe microbead experimental mouse models. SaCas9-*Aqp1* both protected retinal ganglion cells and reduced the levels of IOP and AQP1 in the microbead model. Furthermore, the SaCas9-*Aqp1* system was tested on a postmortem human ciliary body. Transduction of the postmortem human ciliary body with SaCas9-*AQP1* produced low but detectable levels of indel formations in *AQP1* despite the problems in maintaining the ciliary body *ex vivo* (Wu et al., 2020).

Age-related macular degeneration (AMD) is another multifactorial disease where CRISPR-based gene therapy approaches were studied for the treatment of disease symptoms. Age-related macular degeneration (AMD) is the top cause of blindness in people over 55 years of age. It is expected that almost 19 million people will be affected by AMD by the year 2040 in Europe (Colijn et al., 2017). Neovascular AMD is a type of AMD detected by the presence of several large drusen and a choroidal neovascularization complex (Mitchell et al., 2018). Neovascularization results in retinal detachment, fluid accumulation, and scar tissue formation due to aberrant and leaky blood vessel growth in the retina. A treatment for neovascularization has been the focus of researchers around the world. VEGFA is a popular target of inhibition studies as it is one of the major culprits that cause neovascularization (Ferrara, 2016).

Using the CRISPR system, gene expression products can be modulated by producing mutations in the gene loci, which can dramatically reduce expression levels of the Vascular endothelial growth factor A (*VEGFA*). A very recent study used lentivirus-mediated delivery of CRISPR-SpCas9 (mLP-CRISPR) to produce mutations in the *VEGFA* loci for the treatment of AMD. To this end, researchers produced dual-lentivirus (mLP-Cas9) and single-lentivirus (mLP-CRISPR) systems. The mLP-Cas9 dual lentivirus system contains an MS2C modification in the GAG-POL N-terminus and an MS2-stem-loop modification in the Cas9 mRNA to achieve efficient mRNA packaging into the lentivirus. The single lentivirus vector system was found to be more efficient at introducing mutations, as the MS2C modifications in the dual lentivirus system affect transfection efficiency. It was reported that mLP-CRISPR achieved 44% indel formation at the VEGFA loci, resulting in a 35% decrease in VEGFA expression levels in the RPE-choroid-scleral (RCS) tissues of C57BL/6J mice, and 63% reduction of the choroidal neovascularization (CNV) area in the laser-induced wet AMD mice. These results encourage further translational work exploiting gene therapy, especially for non-genetic diseases that affect several million (Ling et al., 2021).

### Cas13 in retinal diseases

CRISPR-Cas13a, previously known as CRISPR-C2c2, is a novel RNA-guided single-stranded RNA (ssRNA)-targeting the CRISPR system that exhibits collateral and on-target ssRNA endonuclease activity (Abudayyeh et al., 2016). Unlike other CRISPR-Cas systems, the Cas13 enzyme demonstrated more efficient RNA editing. The conformational change following the binding of Cas13a protein to the target ssRNA results in a collateral activity, in which non-specific sites nearby the target get cleaved (Varble and Marraffini, 2019). Recently, Konermann et al. identified a new family of Cas13 proteins using bioinformatics tools (Konermann et al., 2018). They mined for CRISPR-Cas sequences in previously sequenced prokaryotic genomes and discovered a new family of type VI CRISPR-Cas13d proteins with a small size (2.8 kb) that is beneficial for AAV packaging. Moreover, the Cas13d family was shown to have no protospacer flanking sequence requirements although they prefer accessible sites and U bases in the target sequence (Konermann et al., 2018). In the same study, CasRx (RfxCas13d) which is one of the orthologs of Cas13d obtained from *Ruminococcus flavefaciens* showed the most efficient knock down capability within the type IV Cas13d family (Yan et al., 2018). CasRx showed robust knockdown of target transcripts with higher editing efficiency in all cases compared to CRISPR-dCas9-inhibitor, shRNA, Cas13a, and Cas13b systems with no reported off-target effects (Konermann et al., 2018). It is also feasible to fuse catalytically inactive dCasRx with the splice factors to enable splicing modulation. This property was tested *in vitro* to exclude exon 10 of the *MAPT* gene in cortical neurons generated from patient-derived human-induced pluripotent stem cells. MAPT encodes for two isoforms of Tau, 3R and 4R. An imbalance in the ratio of 4R/3R due to mutations can cause frontotemporal dementia. dCasRx-mediated splicing exclusion of MAPT exon 10 successfully reduced the 4R/3R tau ratio to nearly healthy levels showing the effectiveness of the system (Konermann et al., 2018).

Effective *in vivo* messenger RNA (mRNA) knockdown experiments have also been performed using CasRx. Zhou and coworkers successfully employed a CasRx tool to knock down RNA binding Ptbp1 protein to initiate a transformation of Müller Glia cells into RGC (Zhou et al., 2020b). Depletion of Ptb1 is shown to induce neuronal fate in cells (Xue et al., 2013). Using this feature the researchers aimed to replenish degenerated RGCs by the conversion of Müller Glia to revert neurodegeneration. Initially, *in vitro* analysis using neural stem cells showed a decrease of approximately 87% in Ptb1 mRNA levels. Afterward, *in vivo* experiments were performed in the mature retina. CRISPR-CasRx system with two gRNAs and AAV-GFAP-GFP-Cre was coinjected into the eyes the of Ai9 mouse model (Rosa-CAG-LSL-tdTomato-WPRE) to label Müller glia (MG). RGC marker expression following CasRx treatment revealed a transformation of MG-to-RGC. In addition, *N-*methyl-D-aspartate (NDMA) injected animals were also used to further explore the conversion of MG-to-RGC in a retinal degeneration environment. MG-to-RGC conversion was observed in the NMDA-injected animals, and immunostaining showed that RGCs made projections into the lateral geniculate nucleus and superior colliculus regions of the brain. A successful improvement in visual responses was indicated in CRISPR-CasRx-mediated knockdown of Ptb1 in treated mice. Thus, RGC cells were recovered successfully in the vision loss model using the CRISPR-CasRx knockdown (Zhou et al., 2020b).

In another study, CRISPR-CasRx was used to target and knockdown the *Vegfa* gene to inhibit CNV in an AMD mouse model and to prevent vision loss. CasRx was delivered *via* AAV into the laser irradiated AMD mouse model. After 7 days, VEGFA protein and *Vegfa* mRNA levels showed a significant reduction in CNV areas. Sequencing data have further confirmed the reduction in *Vegfa* expression (Zhou et al., 2020a). With its high efficiency and specificity, CRISPR-CasRx has received considerable attention among the current CRISPR-Cas systems. It presents a great potential to successfully perform transient knock down of pathogenic genes causing eye diseases.

## Antisense oligonucleotides

Antisense oligonucleotides (AONs) are designer molecules inhibiting mRNA translation and non-coding RNA function through Watson-Crick base pairing, as well as affecting pre-mRNA splicing to correct splicing defects. AONs are single or double-stranded RNA or DNA molecules with modified backbones to increase cellular uptake. Virtually any sequence can be targeted with minimal off-target effects with AONs (Figure 2A). In 1978, AONs were shown to inhibit Rous sarcoma virus RNA function by Stephenson and Zamecnik (1978). Following this discovery, fomivirsen became the first AON drug to be approved by the FDA in 1998. Fomivirsen targeted the CMV major immediate early region (IE2) mRNA to inhibit viral proteins. It successfully prevented cytomegalovirus (CMV) retinitis symptoms in acquired immunodeficiency syndrome (AIDS) patients (Vitravene Study, and Group, 2002). In addition to gene silencing (inotersen, patisiran, volanesorsen), AON therapeutics that are purposed for splicing correction (eteplirsen, casimersen, viltolarsen, nusinersen) were approved by drug regulatory authorities such as FDA and European Medicines Agency (EMA) in the last decade. The success of AON clinical trials and the rapid progress in the field provide great potential for correcting splicing defects and reducing the expression of mutant gene products in IRDs.

AONs have been extensively studied as potential therapeutics against retinal diseases with pathogenic splicing defects and other mutations as well. One of the first AONs that targets an IRD is Ultevursen (QR-421a). It is a therapeutic drug for the treatment of autosomal recessive retinitis pigmentosa (arRP) and usher syndrome (arUSH) which are caused by mutations in exon 13 of the *USH2A* gene. Almost 80% of USH2 patients have mutations in the *USH2A* gene (Stabej et al., 2012). USH2 patients experience hearing loss, later accompanied by vision loss. USH2 is a heterogeneous disease with various deletions, single nucleotide polymorphism (SNP), and aberrant splicing mutations in the USH2 gene (Petit, 2001). Gene replacement with AAV is not possible due to the 15.6 kb size of the USH2A gene (Accession: NM_206933). Thus, ultevursen targets exon 13 of *USH2A* to enable exon skipping to circumvent mutations and produce a smaller yet functional protein product. Ultevursen was tested on USH2 patient-derived induced pluripotent stem cells (iPSCs), USH2A zebrafish, and mouse models with encouraging results (Dulla et al., 2021). Currently, ultevursen is being tested in phase 1, 2, and 3 clinical trials (NCT05158296, NCT03780257, NCT05085964, NCT05176717).

The silencing capabilities of AONs were also used to develop a therapeutic for the treatment of geographic atrophy (GA) caused by AMD. Atrophic AMD, also known as geographic atrophy (GA), is characterized by outer retinal thinning due to the loss of retinal pigment epithelium (RPE) and photoreceptor cells (de Jong et al., 2020). The aberrant complement pathway in GA is reported in the literature. Moreover, complement activation products have been detected at Bruch’s membrane and choriocapillaris as well. The presence of common or rare mutations in genes related to the alternative complement pathway activation is also an important criterion for an inhibitory treatment (de Jong et al., 2020). While a connection between the complement system and GA is indicated, previous studies reported mixed results (Park et al., 2019). Therefore, IONIS-FB-L_*RX*_ has been developed as a ligand conjugated AON drug that inhibits complement factor B (Fb) production to achieve a therapeutic effect. IONIS-FB-L_*RX*_ treatment has successfully reduced Fb levels in animal models as well as humans. Two phase 2 clinical trials are currently recruiting for AMD and primary IgA nephropathy patients separately (NCT03815825, NCT04014335) (Jaffe et al., 2020).

Neovascular glaucoma and keratitis can result in vision loss that can occur because of ischemic central vein occlusion. For the treatment of these diseases, Aganirsen (GS-101) was developed to inhibit the progression of inflammation and neovascularization in the cornea. It is an antisense oligonucleotide delivered in the form of an eye drop. Aganirsen targeted the insulin receptor substrate I (IRS-I) to inhibit neovascularization by downregulating vascular endothelial growth factor (VEGF) (Al-Mahmood et al., 2009). It was shown in preclinical studies that the topical treatment of aganirsen is non-toxic in animals and the treatment only targets aberrant neovascularization while having no effect on normal vascularization (Cloutier et al., 2012). Aganirsen was reported to significantly reduce corneal neovascularization area and reduced the transplantation rates in phase II and III clinical trials (Cursiefen et al., 2009, 2014). However, no change in visual acuity was observed. It is currently underway a phase II/III clinical trial with 333 subjects (NCT02947867).

For the treatment of LCA10, which is caused by the c.2991+1655A > G (p.Cys998) mutation in the *Cep*290 gene, an RNA-based AON drug, Sepofarsen (QR-110), was developed. The Sepofarsen targets the mutated CEP290 pre-mRNA and restores normal mRNA splicing. Compared to the aforementioned genome editing methods that are targeting CEP290 where the mutation is removed by a Cas9 enzyme, the AON approach provides a safer and transient alternative as no genome editing takes place. Sepofarsen showed a significant increase of wt *CEP290* in patient iPSC-derived retinal organoids (Dulla et al., 2018). Following promising preclinical results, a phase I/II clinical trial was performed (NCT03140969). Currently, there are two active phase II/III clinical trials for this drug (NCT04855045, NCT03913143).

Accumulation of mutant RHO in rod photoreceptors has a cytotoxic effect and reducing the amount of mutated RHO products should reduce the severity of RP (Athanasiou et al., 2018). In pursuit of this hypothesis, QR-1123 (NCT04123626), an AON intravitreally injected drug has been designed for autosomal dominant RP, which causes permanent vision loss. This drug promotes RNase H-directed cleavage of the mutated (P23H) RHO mRNA and enhances the activity of wild-type rhodopsin protein. This was an outstanding approach that targets numerous mutations at once to cure severe retinal diseases, therefore, QR-1123 has received a fast-track designation from FDA to provide the potential drug to people who are in urgent need. The phase I/II clinical study started with 35 patients for a 12-month treatment period. QR1123 has been a promising approach for silencing mutant RHO mRNA to enhance vision and prevent blindness (NCT04123626) (Biasutto et al., 2019).

## Base editing and its applications in retinal diseases

Base editing is a recently developed gene editing approach that enables the direct conversion of one DNA or RNA base pair to a specific base pairing using base-editing enzymes fused to a CRISPR-Cas9 protein (Figure 1E). Base editors produce precise point mutations/corrections without inducing double-stranded DNA breaks (DSBs). In contrast to traditional gene editing techniques that utilize DNA repair mechanisms such as non-homologous end joining (NHEJ) and homology-directed repair (HDR), base editors provide more efficient point mutations with less undesired and/or harmful by-products (Suzuki et al., 2016; Kim et al., 2017; Suh et al., 2021).

Base editors can be categorized into two main groups: DNA and RNA base editors targeting DNA, and RNA, respectively. There are two main DNA base editors: Cytosine base editors (CBEs) and adenine base editors (ABEs). CBEs use cytidine deaminase to convert cytosine to uracil which facilitates the conversion of C•G to T•A base pair. As with the CBEs, ABEs use adenine deaminase to convert adenine to inosine, which pairs with cytosine (Kim et al., 2017; Komor et al., 2017). This method enables the permanent correction of a wide range of point mutations and was first utilized by Komor et al. by using a cytidine deaminase enzyme. Moreover, Cas9 nickase was shown to have a better editing efficiency compared to dCas9 and was incorporated into a new generation of base editors (Komor et al., 2016).

Base editors have tremendous therapeutic potential and have been used in proof-of-concept studies of various diseases including inherited retinal disorders as the most pathogenic genetic variants arise from point mutations (Levy et al., 2020; Fry et al., 2021; Suh et al., 2021). However, delivery of base editors already poses a problem as the total size of the whole construct exceeds far beyond the packaging capacity of AAVs. This size restriction was solved by delivering base editors split into dual AAV vectors (Levy et al., 2020). Researchers split base editors into amino-terminal and carboxy-terminal fragments and obtained the full-length protein through intein-mediated protein *trans*-splicing. PHP.B and Anc80 AAV capsids were used to utilize efficient rod photoreceptor targeting. Split-base editors targeting the *Dnmt1* (*DNA Methyltransferase 1*) gene were delivered by dual AAVs in a rod photoreceptor reporter mouse line with a silent mutation in the *Dnmt1* locus. Researchers reported that both PHP.B and Anc80 AAVs achieved similar transduction efficiencies in rod photoreceptors at the same dose (5 × 10^9^ vg). Both PHP.B-CBE and Anc80-ABE resulted in significant editing events; however, CBE had high off-target indel frequency. Split-CBE and -ABE delivered by dual AAVs achieved similar results to single vector solutions.

People suffering from LCA2 with biallelic mutations in the *RPE65* gene are normally treated by sub-retinal voretigene neparvovec. However, studies reveal that despite initial visual enhancement, retinal degeneration lingers in the long term with gene replacement therapy (Gardiner et al., 2020). A proof-of-concept study by Suh et al. (2021) was proposed as a possible strategy to advance the current gene editing technology for LCA2 disease. Using ABE delivered by lentivirus, they corrected a non-sense mutation in the exon 3 of the *RPE65* gene in the rd12 mice, which is an autosomal recessive RP and LCA mouse model (Pang et al., 2005). Deep sequencing at 5 weeks post-injection revealed a 32% rescue of RPE65 protein and as much as 29% correction of the non-sense mutation. Overall, base editors are capable of targeting and correcting pathogenic SNPs that cause IRDs.

While base editors offer an effective alternative to gene replacement therapies, RNA base editors offer a safer alternative. Since RNA mutations are transient and reversible, this approach is considered safer compared to DNA-base editing. In addition, RNA base editing reduces the risk of creating permanent off-target changes since it occurs at a posttranscriptional level. In general, two genomic transition variants of G•A and T•C can be corrected by RNA base editors (Fry et al., 2020). A large number of RNA base editors can be delivered *via* a single AAV. They showed promising *in vivo* potential using adenosine deaminase acting on RNA (ADAR) on two mice models of human diseases, including *mdx* mouse model of DMD and *spf^ash^* mouse model of Ornithine transcarbamylase (OTC) deficiency. More than half of the pathogenic mutations causing recessive IRD can be corrected using RNA base editors, indicating their importance for retinal gene therapy (Katrekar et al., 2019; Fry et al., 2021).

## Prime editing approach

Prime editing is a gene editing technique that precisely edits DNA mutations without requiring HDR, NHEJ, or DSBs (Figure 2F). It enables any targeted small insertions and deletions, and all 12 possible base-to-base conversions, and their combinations. Prime editing allows programmable point mutations by fusing a reverse transcriptase (RT) to dCas9 and targets DNA by prime editing guide RNA (pegRNA). It was developed by Anzalone et al. (2019) as a “search and replace” genome editing method to advance the base editing approach for all possible transition and transversion mutations. There are four types of prime editors (PEs) named PE1, PE2, PE3, and PE3b. PE1 and PE2 consist of a Cas9 nickase fused to an engineered RT, but PE2 is more efficient and compatible with shorter primer binding site sequences. PE3 and PE3b comprise an extra sgRNA that enhances the editing efficiency by targeting the non-edited strand for nicking (Anzalone et al., 2019; Song et al., 2021).

Jang et al. applied the prime editing approach as a proof-of-concept study for IRD (Jang et al., 2021). They showed that PE2 can perform a highly specific, targeted, and efficient correction of a non-sense mutation in the rd12 mouse model. A *trans-*splicing AAV2 vector was used to deliver the PE2 system that will correct the non-sense mutation in exon 3 of the RPE65 gene. The split AAV2 system (AAV-PE2) achieved 23% transduction of the RPE layer while retaining a 6.4% editing efficiency throughout the RPE layer in rd12 mice. PE2 performed highly specific editing with no unintended mutations adjacent to the editing site. Moreover, they showed that correcting the non-sense mutation using PE2 improved visual acuity in rd12 mice. Prime editing showed a robust, safe, and specific correction of an IRD-causing mutation with higher efficiency compared to their previous approach where they corrected the non-sense mutation with the HDR repair of CRISPR-Cas9-mediated DSB (Jo et al., 2019).

## Artificial transcription factors

The decoding of a cell’s genome is controlled by transcription factors (TFs) that are devoted to their molecular functions for the survival of the cell. Transcription factors have several functions that are crucial for a cell such as controlling the regulatory element networks, regulation of transcription, mobilization of coactivator and corepressors, as well as interactions with other TFs to integrate multiple signaling networks (Khalil et al., 2012). Mimicking the innate properties of TFs is already an attractive tool for gene regulatory therapeutic approaches. This could be achieved by generating artificial transcriptional factors (ATFs) which are designer TFs that enable robust control of eukaryotic gene expression (Pandelakis et al., 2020). ATFs benefit from the sequence-specific DNA binding properties of zinc finger protein (ZFP), TALE, or dCas9. These are commonly fused to one or a combination of activators VP64, RTA, and p65 which allows the activation or overexpression of the target gene. Additionally, the VPR activator that is produced by combining VP64, p65, and RTA domains is commonly fused to ATFs for strong upregulation of a target gene (Chavez et al., 2015; Zhang et al., 2019; Scott et al., 2021). On the other hand, fusing ATFs with repressors like SID and KRAB can significantly suppress the target gene expression (Gilbert et al., 2013; Konermann et al., 2013). It is also possible to recruit transcriptional activators/repressors, without producing dCas fusion proteins, using a multitude of ways. A modified gRNA containing protein-interacting aptamers that recruit bacteriophage coat proteins such as MS2 was used to develop a dCas9-synergistic activation mediator (SAM) (Konermann et al., 2015). Another similar approach was used to develop dCas9-SPH, where the transcriptional regulatory domain is bound to an scFv domain (Zhou et al., 2018). Therefore, using these novel tools, it is now feasible to establish a synthetic gene regulatory network and reprogram gene regulation.

### CRISPR-dCas9 and its applications

The gRNA-specific DNA binding capabilities of CRISPR-Cas9 systems make them attractive targets for gene regulatory applications. For that CRISPR-dCas9 was developed as a tool for transcriptional regulation of gene expression without modifying the genomic DNA. The enzymatic activity of Cas9 was mutated to produce dCas9 (Qi et al., 2013). The fusion of activator or repressor domains to a dCas9 that is guided by a gRNA enables the regulation of target gene transcriptional machinery. These properties also make it possible to manipulate multiple target genes simultaneously in a reversible manner. With the fusion of different activators/inhibitors and inducible domains to dCas9, one can establish gene regulatory networks by turning genes on and off. Inducible domains such as gibberellin and abscisic acid can be fused to different CRISPR-dCas9 variants, for multiplexing purposes, to produce synthetic networks. Furthermore, these systems can be multiplexed using more than one guide RNA simultaneously. dCas9-based artificial transcription factors are ideal for producing simultaneous activation or inhibition of multiple target genes with minimal leaky expression (Gao et al., 2016; Zhou et al., 2018).

In a recent study, a split CRISPR-dCas9-VPR system was used for the transactivation of functionally equivalent genes to restore the function of rods. M-opsin and S-opsin are cone opsins that are homologs of Rhodopsin. Although they have different expression patterns, their physical properties are remarkably similar. M-opsin and S-opsin were upregulated in a rhodopsin haploinsufficiency mouse model that mimics RP (Böhm et al., 2020). Interestingly, M-opsins compensated for rhodopsin deficiency in rod photoreceptors. This slowed down the rate of degeneration, improved the function of rod cells, and induced a cone-like phenotype due to M-opsin overexpression in rods. This intriguing proof-of-concept study opened up new treatment possibilities and a modality for rescuing mutant phenotypes using functionally equivalent genes.

### TALE and TALEN

TALEs are used in effective and targeted genome modifications due to their specific DNA binding properties. The addition of a nuclease provides a precisely targeted cutting location in DNA. TALEs, also known as Type III effectors, were first identified in *Xanthomonas* bacteria. They typically contain 34 amino acid repeats that are located in the transcriptional activation domain (AD) of the protein. These repeats are highly conserved between TALEs except for the two hypervariable residues that facilitate the target DNA sequence recognition. These residues, named repeat variable di-residues (RVDs), can recognize more than one nucleotide with the same amino acid combination. Moreover, TALEs can virtually target any sequence with at least 11.5 repeats. They are highly specific and able to discriminate between methylated and non-methylated states of nucleotides with different RVD combinations. TALE recognition of the target DNA sequence can activate or repress gene expression through TALE-mediated transcription complexes or transcription regulatory protein fusion to TALE. Highly specific binding to virtually any DNA sequence makes TALEs great tools for the production of artificial transcription factors (Sanjana et al., 2012; Zhang et al., 2014; Becker and Boch, 2021). In addition, TALENs, are modified TALE proteins that are fused with a non-specific *FokI* endonuclease domain (Sanjana et al., 2012). TALENs form a dimer and introduce DSB after target DNA recognition, making them ideal for gene knockout experiments.

TALE-based artificial transcription factors (T-ATFs) are produced from the DNA targeting domain of TALE and an effector domain. T-ATFs target the transcription start site of genes to modulate their expression. It was shown that T-ATF fused to the VP64 activation domain successfully upregulated *SOX2* and *KLF4* genes that are crucial for cellular reprogramming (Zhang et al., 2011). T-ATFs can target closed chromatin structures, which should make cell reprogramming more accessible (Perez-Pinera et al., 2013). A proof-of-concept study showed successful genetic upregulation of the *VEGF-A* gene with a T-ATF-VP64 construct targeted at the DNase I hyper-sensitive site (HSS) of the *VEGF-A* gene. They achieved significantly increased levels of VEGF expression using VP64-TALE and p65-TALE on their own and together as well (Maeder et al., 2013). The robust transcriptional regulation provided by T-ATF targeting *VEGF-A* indicates that replacing the VP64 domain with a repressor, such as the KRAB domain, would make it a potential therapeutic for neovascular glaucoma and wet AMD. T-ATFs provide similar transcriptional regulation potency to Zinc Fingers (ZFs) while being more accessible, making them ideal for gene therapy research.

### Zinc finger artificial transcription factors

ZFPs were first identified in 1985 soon after the discovery of meganucleases. A protein isolated from the *Xenopus laevis* oocyte containing zinc-binding domain repeats was found to be required for the 5S rRNA transcription by Miller, McLachlan, and Klug, which was the first reported ZFP (Miller et al., 1985). Lately, it was found that ZFPs are the largest transcription factor family in the human genome. The crystal structure of the three-finger ZFP Zif268 showed that each finger connects to a 3-bp subsite on DNA (Pavletich and Pabo, 1991). This inspired various scientists to generate custom ZFPs from these modules. Different combination of modules results in a ZF that binds to different specific sequences which are called modular assembly. Although *in silico* design of ZFs are possible, context-dependent effects related to a specific ZF and its target DNA sequence can result in suboptimal ZF effectiveness (Bhakta and Segal, 2010). Therefore, screening for a combination of 6-8 ZF modules to target 18–24 bp DNA sequences is a common practice (Bippes et al., 2022). This also makes the ZF approach quite labor-intensive, unlike CRISPR-cas9 and TALE.

ZFs can be used either as DNA modifying enzymes by fusing with a nuclease (Kim et al., 1996) or as an artificial transcription factor by fusing with transcriptional activators. The first designs of ZFNs exploited *FokI* endonucleases fused to ZFs. An efficient DSB was achieved by designing ZFN dimers targeting specific DNA sequences (Paschon et al., 2019). Each ZFN forming the ZFN dimer interacts with the target sequence precisely to enable the alignment of two *FokI* endonuclease domains and to produce a DSB. This made ZFN a common tool to knockout several genes both *in vivo and in vitro.*

An important advantage of ZFs over other nucleases or artificial transcription factors is that they are of human origin. Overexpression of CRISPR-Cas and TALE proteins following delivery has the potential to generate an immune response in target cells as they originate from bacteria. Inflammation and other complications arising due to immunogenicity further cause cell death in the treated region (Li et al., 2020; Mehta and Merkel, 2020). The risk of these undesired outcomes can be overcome with ZF owing to the human origin of the therapeutic ZFNs and ZF-based artificial transcription factors. The small size of ZFs also gives them tremendous advantages. This property was beneficial for the recent advancements in delivering ZF-based ATFs. Despite the vast benefits like minimal size and immunogenicity, the usage of ZF as ATFs is very limited.

Mutations in the rhodopsin gene are known to cause autosomal dominant and recessive forms of RP. Due to strict ER regulation on the rhodopsin protein folding, even point mutations can have a major impact on rod photoreceptor health. Thus, the ideal treatment for RP should inhibit mutant Rho expression while promoting wild-type Rho expression (Athanasiou et al., 2018).

Mutations in the rhodopsin gene are known to cause autosomal dominant and recessive forms of RP. Due to strict ER regulation on the rhodopsin protein folding, even point mutations can have a major impact on rod photoreceptor health. Thus, the ideal treatment for RP should inhibit mutant Rho expression while promoting wild-type Rho expression (Athanasiou et al., 2018). For Usher syndrome (Overlack et al., 2012) and RP (Greenwald et al., 2010), ZFNs were used as a possible treatment, however, they required homologous recombination to replace the mutant gene. It should be noted that exploiting homologous recombination is very disadvantageous in photoreceptors as they are non-dividing cells. In a more recent study, Mussolino and colleagues employed ZF-ATFs for the treatment of RP. They developed a ZF-RHO-KRAB system that inhibits all rhodopsin expression. As RP is a very heterogenic disease with copious mutations, they developed a treatment strategy by repressing all Rho expression and replacing the Rho gene with the WT counterpart afterward. Their initial results suggest that repressing Rho expressions in RP mouse models can protect rod photoreceptors and preserve visual function (Mussolino et al., 2011). Furthermore, they tested this system on early postnatal murine retinas and showed that treated retinas had a thicker outer nuclear layer (ONL) and better visual function compared to untreated retinas (Marrocco et al., 2021). This ultimately shows that the treatment of early-onset IRDs using ZF-ATFs is possible without major safety concerns and that ZF-ATFs can achieve efficient neuroprotection in the retina.

A pioneering study was performed showing the *in vitro* efficiency of ZFs as ATAs and their *in vivo* deliverability. For the last 20 years, protein delivery has been a big challenge to be resolved. Through trial-and-error, delivery efficiency was discovered to be the limiting problem. Typically, %1 of the delivered product can enter cells (Kaplan et al., 2005). The method of preference was a fusion protein of the *trans-*activator of transcription (TAT) protein, an HIV protein, which enhances the efficiency of viral transcription. TAT induces endosomal uptake of the fused protein. However, the inefficient release of the TAT from the endosomal membrane traps the protein and results in inefficient delivery of the target protein, and degradation. Albert Neutzner and his group overcame this problem using digestion enzymes existing within the endosome (Bippes et al., 2022). This novel delivery method benefits from the cathepsin proteases. A TAT peptide fusion protein was produced after screening for the efficient cathepsin B cut sites. The cathepsin B cut side allows the release of the target protein within the endosome and the escape of the protein.

This was demonstrated by a ZF-SID protein which was designed to inhibit endothelin receptor A (ETRA) expression. ETRA was chosen since its expression is involved in vasoconstriction and connected to pulmonary hypertension causing ocular diseases, such as retinal vein occlusion. Researchers screened for a ZF-SID that would inhibit ETRA1 expression *in vitro* which was quite labor-intensive. The resulting ZF-SID was fused with a TAT peptide, including the Cathepsin B cut site and NLS sequence, and tested *in vitro* which showed successful protein delivery and knockdown of ETRA expression. This was further followed by *in vivo* experiments. Uptake of ZF-SID in corneal, retinal, and ciliary body cells was shown in a pig model after intravitreal delivery. An efficient uptake that persisted for 96 h was achieved, showing a transient nuclear localization most likely sufficient for the treatment of several disorders. This fascinating result was the first efficient protein delivery method to be established and will have several applications for retinal diseases in the future including transient gene therapies. This was also one of the first demonstrations of the reversible effects of ATFs both *in vitro* and *in vivo*.

## Discussion

Approximately 80% of retinal gene therapy trials are gene replacement therapies that are complementing the loss-of-function mutations. Despite the beneficial effects of gene replacement approaches, the therapeutic outcome might be limited due to uncontrolled overexpression of the target gene. Moreover, there are several cases where delivery of a single variant of cDNA is unlikely to be sufficient to restore full gene function. Size restriction of the AAV, complexity of the target gene such as having multiple splicing events, subsequent proteolytic processing, or pathogenic mutations limit these gene therapy attempts. Therefore, several retinal diseases are out of the scope of gene replacement therapies and needs to be targeted with more complex gene editing and gene regulatory approaches.

With the recent improvements in applications of CRISPR-Cas9 and associated tools, it is now feasible to correct the mutant genes, which will bypass the aforementioned problems. Despite a tremendous number of proof-of-concept studies using CRISPR Cas9 and related tools, there is only a handful of clinical trials that target the mutant genes due to efficiency and safety issues. However, CRISPR Cas9 itself is already an effective tool where exact corrections or additional deletions are of less importance. These could be intronic mutations (Ruan et al., 2017; Maeder et al., 2019), complete elimination of the pathogenic allele, or a specific diseases condition like VEGFA inactivation which is an important target for the wet AMD condition (Ling et al., 2021).

Despite all the recent promising applications of gene editing tools, off-target effects including genome integration are additional challenges for gene therapy applications. It is normally accepted that AAVs encounter very low integration rates into the genome. However, an intriguing study demonstrated the unexpected acceleration of AAV genome integration. Surprisingly AAVs integrated at the CRISPR cut sites of all on-target genes with high frequencies (Hanlon et al., 2019). This phenomenon needs to be considered together with off-target gene modifications that are introduced with CRISPR-Cas9 approaches. To minimize the off-target modifications, several transient base-editing approaches were developed. These novel approaches use nanoparticles and extracellular vesicles that deliver Cas9 protein and thus limit the availability of Cas9 for editing (Gee et al., 2020; Wei et al., 2020). Reduced off-target effects are already observed with these transient base-editing methods (Gee et al., 2020; Wei et al., 2020; Ling et al., 2021). A recently developed protein delivery method using cathepsin sites for endosomal escape and ATFs could also be an intriguing option for transient base editing (Bippes et al., 2022).

CRISPR-Cas9 is ideal for gene/exon knockout, however, there are pathogenic point mutations that need to be corrected with a more precise toolset. Base editors that use CRISPR-Cas9-nickases can perform this highly specific editing work as shown in the rd12 mouse model (Suh et al., 2021). In addition to base editing, prime editing brings great potential for rescuing point mutations that cause IRDs and its efficacy was shown in the rd12 mouse model as well (Jang et al., 2021). Yet, it remains that these gene editing tools rely on the CRISPR-Cas system that is prone to off-target editing, and are restricted by the PAM sequence availability. The development of CRISPR-CasRx allowed researchers to address these issues as CasRx does not require a flanking recognition sequence such as PAM, targets RNA and performs better than dCas9 or shRNA. Moreover, CasRx can be packaged into a single AAV which is not possible with base and prime editing (Konermann et al., 2018; Liu et al., 2020). These CRISPR-based editing tools provide a diverse approach to the gene editing field, however, there are critical aspects to be considered when adapting them into a translational study. Considerations should be made into the target tissue types, delivery strategies, as well as optimization of the process from *in vitro* to *in vivo* assessments to preclinical and future clinical applications.

Even though there are tremendous developments in base editing approaches, the size of constructs together with the low efficiency are still ongoing problems. In that sense, AON therapy which is used to deploy pre-mRNA splicing or downregulation to slow down the progress of ocular diseases such as LCA, Usher syndrome, or RP is an ideal solution (Xue and MacLaren, 2020). Implications of both naked AONs and AAV integrated AONs already indicated successful improvements in CEP290 protein levels for the treatment of LCA10 with the common c.2991+1655A > G mutation in the CEP290 gene (Xue and MacLaren, 2020; Leroy et al., 2021). Depending upon the success of the clinical phase II/III trials of Sepofarsen, it can be the first AON therapeutics that is approved by the FDA. CRISPR-Cas9 system with dual AAV genome integration has been suggested as another approach for the efficient treatment of LCA10 (Ruan et al., 2017; Rasoulinejad and Maroufi, 2021). Despite its successful implication for the treatment of IVS26 splice mutation in LCA10, the long-term effects regarding bacterial Cas9 protein as well as the off-target effect are still a concern (Ruan et al., 2017). Since AONs are effective at the pre-mRNA level, they prevent off-target and inflammatory toxic effects (Xue and MacLaren, 2020). In addition, they can be delivered either *via* intravitreal injections. The only burden in the delivery method of AONs is repeated regular injections (up to 6 months) to achieve an acceptable dose (Leroy et al., 2021). Despite the efficacy of AON-based gene therapies, heterogeneous IRDs are still a major obstacle to designing oligonucleotides because a specific oligonucleotide for each mutation should be designed (Huang et al., 2017; Xue and MacLaren, 2020).

Luxturna treatment successfully improved vision in patients but could not stop the ongoing degeneration (Cideciyan et al., 2013; Jacobson et al., 2015), resulting in significant neuronal and again visual loss. There is thus an emerging need for effective neuroprotective therapies and complementary regenerative approaches for a full recovery that stops degeneration and restores vision loss. Neuroprotective approaches for overexpression of several factors have also been studied for decades (Leibinger et al., 2009; Rhee et al., 2013). However, dose adjustments for neuroprotective factors *in vivo* are often not successful (Birch et al., 2016). Excess overexpression usually leads to toxicity or loss of functionality (Graham et al., 2005) (Liang et al., 2001; Birch et al., 2016). In that respect, a novel and promising technology could be the application of artificial transcription factors using the sequence-specific DNA-binding properties of TALE, dCas9, or Zinc fingers. Using different combinations of activators, ATFs control and upregulate the level of gene expression. This could also overcome the dosage problem as this property of ATFs allows upregulating the gene of interest in a more controlled manner. Moreover, multiplex targeting of neuroprotective factors could be achieved to establish combinatorial neuroprotective therapies, which would yield more efficient therapies (Leibinger et al., 2009).

Since the fusion of different activators as well as the target site on the target promoter determines the dosage of the target gene product, ATFs could also be ideal tools for haploinsufficiency conditions where a single copy wild-type protein does not yield sufficient amounts of protein for normal function. The output of mRNA and thus proteins from the wild-type copy could be enhanced by different activators, which can be adjusted to yield sufficient protein from the wild-type copy. More tests to enhance the output needs to be done on the specific promoter region, the choice of the activator protein, and the choice of the system.

ATFs can also be used as negative regulators of gene expression. This is especially important for dominant negative retinal diseases. Fusion of ATFs with negative regulators like SID or KRAB suppresses the target gene. Depending on whether it is a point mutation or an indel, ATFs can either target the specific mutant site or knock down both wild-type and the mutant copy of the target gene. In the latter case, simultaneous delivery of wild-type cDNA as a dual therapeutic approach is also necessary. ATF-based overexpression and repression also allow the establishment of synthetic gene regulatory networks *in vivo* which might be an ideal tool to study more complex approaches like retinal regeneration *in vivo*.

Although several gene editing tools are capable of achieving similar results in proof-of-concept studies, their *in vivo* efficiencies vary greatly. For example, there are gene replacement and gene correction studies for the treatment of RPE65-related LCA and RP. VN is an already established FDA-approved therapy, however, its benefits over the long term are still debated with complications arising following injections despite an improvement in visual acuity (Gange et al., 2022; Reichel et al., 2022). While base editing and prime editing techniques are safer, more precise, and show high editing frequencies, they still have the risk of off-target genome editing and require split AAV (Jang et al., 2021) or lentiviral delivery (Suh et al., 2021) due to size constraints. Similar to RPE65 gene therapies, there are also several gene therapy approaches for VEGFA inhibition for the treatment of neovascularization, each providing different advantages and disadvantages (Zhou et al., 2020a; Ling et al., 2021). A CRISPR-Cas9 proof-of-concept study shows promise for the inhibition of VEGFA, however, it requires lentiviral delivery and still have a low risk of off-target mutation (Ling et al., 2021). The novel CRISPR-CasRx inhibition of *Vegfa* mRNA eliminates the risk of off target genome editing and offers a great treatment opportunity due to its high efficiency. However, CasRx treatment requires further research on safety *in vivo* (Zhou et al., 2020a). AON treatment also provides a transient approach and have no genome editing risks similar to CasRx, however, it requires several rounds of intravitreal injections unlike the AAV delivered gene regulatory tools.

Given all the findings, gene therapy will have a valuable impact on patients who suffers from IRD. It offers to cure permanent blindness with a single treatment and thus it is seen as a breakthrough in medicine compared to conventional treatment techniques. However, as for all rare genetic diseases, possible risks may develop throughout the long-term applications and these should be foreseen and avoided with the improvements in the therapy. Overall, several novel applications have aimed to provide safety and functionality whilst improving vision or preventing vision loss.

## Author contributions

CA, HA, MT, and ZK conceived and designed the manuscript. HA, CA, MT, FO, FA, ZK, and MA wrote the manuscript. All authors contributed to the article and approved the submitted version.

## Footnotes

1
clinicaltrials.gov
